# Identification of a novel uterine leiomyoma GWAS locus in a Japanese population

**DOI:** 10.1038/s41598-020-58066-8

**Published:** 2020-01-27

**Authors:** Kensuke Sakai, Chizu Tanikawa, Akira Hirasawa, Tatsuyuki Chiyoda, Wataru Yamagami, Fumio Kataoka, Nobuyuki Susumu, Chikashi Terao, Yoichiro Kamatani, Atsushi Takahashi, Yukihide Momozawa, Makoto Hirata, Michiaki Kubo, Nobuo Fuse, Takako Takai-Igarashi, Atsushi Shimizu, Akimune Fukushima, Aya Kadota, Kokichi Arisawa, Hiroaki Ikezaki, Kenji Wakai, Taiki Yamaji, Norie Sawada, Motoki Iwasaki, Shoichiro Tsugane, Daisuke Aoki, Koichi Matsuda

**Affiliations:** 1 Laboratory of Genome Technology, Human Genome Center, Institute of Medical Science, University of Tokyo, Tokyo, Japan; 2 Keio University School of Medicine, Department of Obstetrics and Gynecology, Tokyo, Japan; 3 Department of Clinical Genomic Medicine, Graduate School of Medicine, Dentistry and Pharmaceutical Sciences, Okayama University, Okayama, Japan; 4 International University of Health and Welfare School of Medicine, Department of Obstetrics and Gynecology, Chiba, Japan; 5 RIKEN Center for Integrative Medical Sciences, Kanagawa, Japan; 6 Department of Genomic Medicine, Research Institute, National Cerebral and Cardiovascular Center, Osaka, Japan; 7 Tohoku Medical Megabank Organization, Tohoku University, Sendai, Japan; 8 Iwate Tohoku Medical Megabank Organization, Iwate Medical University, Iwate, Japan; 9 Department of Health Science, Shiga University of Medical Science, Shiga, Japan; 10 Department of Preventive Medicine, Institute of Biomedical Sciences, Tokushima University Graduate School, Tokushima, Japan; 11 Department of General Internal Medicine, Kyushu University Hospital, Fukuoka, Japan; 12 Department of Preventive Medicine, Nagoya University Graduate School of Medicine, Aichi, Japan; 13 Division of Epidemiology, Center for Public Health Sciences, National Cancer Center, Tokyo, Japan; 14 Center for Public Health Sciences, National Cancer Center, Tokyo, Japan; 15 Laboratory of Clinical Genome Sequencing, Department of Computational Biology and Medical Sciences, Graduate School of Frontier Sciences, University of Tokyo, Tokyo, Japan

**Keywords:** Computational biology and bioinformatics, Genetics, Molecular biology, Diseases, Health care, Molecular medicine, Pathogenesis, Risk factors

## Abstract

Uterine leiomyoma is one of the most common gynaecologic benign tumours, but its genetic basis remains largely unknown. Six previous GWAS identified 33 genetic factors in total. Here, we performed a two-staged GWAS using 13,746 cases and 70,316 controls from the Japanese population, followed by a replication analysis using 3,483 cases and 4,795 controls. The analysis identified 9 significant loci, including a novel locus on 12q23.2 (rs17033114, P = 6.12 × 10^−25^ with an OR of 1.177 (1.141-1.213), LINC00485). Subgroup analysis indicated that 5 loci (3q26.2, 5p15.33, 10q24.33, 11p15.5, 13q14.11) exhibited a statistically significant effect among multiple leiomyomas, and 2 loci (3q26.2, 10q24.33) exhibited a significant effect among submucous leiomyomas. Pleiotropic analysis indicated that all 9 loci were associated with at least one proliferative disease, suggesting the role of these loci in the common neoplastic pathway. Furthermore, the risk T allele of rs2251795 (3q26.2) was associated with longer telomere length in both normal and tumour tissues. Our findings elucidated the significance of genetic factors in the pathogenesis of leiomyoma.

## Introduction

Uterine leiomyoma is one of the most common gynaecologic benign tumours. Its estimated lifetime risk is 30-50% in Japan^1^ and 70-80% in European populations^2,3^. Although leiomyoma is a benign neoplasm, patients exhibit many types of symptoms, such as vaginal bleeding, pelvic pain, or infertility^4^. Over 600,000 hysterectomies were performed per year in the USA due to leiomyomas, and 9.4 billion dollars for annual medical expenses were needed in the USA^5^. Therefore, leiomyomas are gaining much attention in health economics.

Leiomyoma growth is stimulated by sex steroids, such as oestrogen and progesterone, and several aetiological factors, such as age, early age at menarche, obesity, and parity, are linked to an increased leiomyoma risk^2,6–11^. In addition, there is some evidence for a genetic component of disease predisposition. African American women have a higher risk than European-American women^2^, and first-degree relatives of affected women have a 2.5-fold greater risk than population average^12^. We also reported that a positive family history is associated with a higher leiomyoma risk among Japanese individuals (odds ratio of 5.496 (5.061–5.969))^13^.

There have been six published genome-wide association studies (GWAS) of uterine leiomyoma, and some common single nucleotide polymorphisms (SNPs) associated with leiomyoma risks at 1p36.12 (*CDC42/WNT4*)^14–17^, 1q24.3 (*DNM3*)^16^, 2p25.1 (*GREB1*)^14,15,17^, 2p23.2 (*BABAM2*)^17^, 3p24.1 (*NEK10*)^14^, 3q26.2 (*TERC/LRRC34*)^15,17^, 3q29^15^, 4q12 (*SCFD2/LNX1/PDGFRA*)^14,15,17^, 4q13.3 (*SULT1B1/SULT1E1*)^14,15,17^, 4q22.3(*PDLIM5*)^17^, 5p15.33 (*TERT*)^14,15,17^, 5q35.2 (*ZNF346*)^15,17^, 6p21.31(*GRM4/HMGA1*)^17^, 6q25.2 (*SYNE1/ESR1*)^14–17^, 9p24.33 (*KANK1*/*DMRT1/ANKRD15/LOC105375949*)^14–17^, 10p11.22(*ZEB1/ARHGAP12*)^17^, 10q24.33 (*OBFC1*)^14–18^, 11p15.5 (*SCGB1C1/BET1L/SIRT3/RIC8A*)^14–18^, 11p14.1(*FSHB*)^17^, 11p13 (*WT1/PDHX/CD44*)^14–17^, 11q22.3 (*ATM/C11orf65/KDELC2*)^14,15,17^, 12q13.11 (*SLC38A2/LOC100288798*)^15,17^, 12q15(*PTPRR*)^17^, 12q24.31 (*PITPNM2*)^17^, 13q14.11 (*LINC0/FOXO1*)^14,15,17^, 16q12.1 (*HEATR3/SALL1*)^15,16^, 17p13.1 (*TP53*)^14–17^, 20p12.3 (*MCM8*)^14,17^, 20q13.13(*LOC105372640)*^16^, 22q13.1 (*TNRC6B/CYTH4*)^14–19^, Xq13.1 (*TEX11/MED12*)^15,17^, and Xq26.2 (*RAP2C*)^15,17^. In addition, uterine leiomyoma harbors genetic alteration of some driver genes including *MED12* mutations, biallelic inactivation of *FH*, and *HMGA2* rearrangements^20–22^. However, only a portion of the genetic variations of uterine leiomyoma could be explained. In a previous study using 1,607 Japanese patients, our group identified three risk loci for uterine leiomyomas on 10q24.33, 11p15.5, and 22q13.1. To gain insights into the genetic causes of uterine leiomyoma, here we performed a GWAS using 5,720 patients of Japanese ancestry.

## Results

### Genome-wide association study of uterine leiomyoma

A total of DNA samples from 5,720 uterine leiomyoma patients and 17,492 controls were analysed in stage 1 (GWAS screening stage 1). All samples were genotyped using the Illumina OmniExpressExome or OmniExpress and HumanExome BeadChip in a previous analysis^23^. After performing a standard quality-control procedure, we conducted genome-wide imputation and obtained the genotyping results of 7,521,072 SNPs. A Manhattan plot of stage 1 analysis is shown in Supplemental Fig. S1. We found four loci (1p36.12, 10q24.33, 11p15.5, 22q13.1) with a genome-wide significance threshold. The genomic inflation factor λ was calculated to be 1.18 and 1.02 (λ_1000_) (Supplemental Fig. S2)^24^.

**Figure 1:**
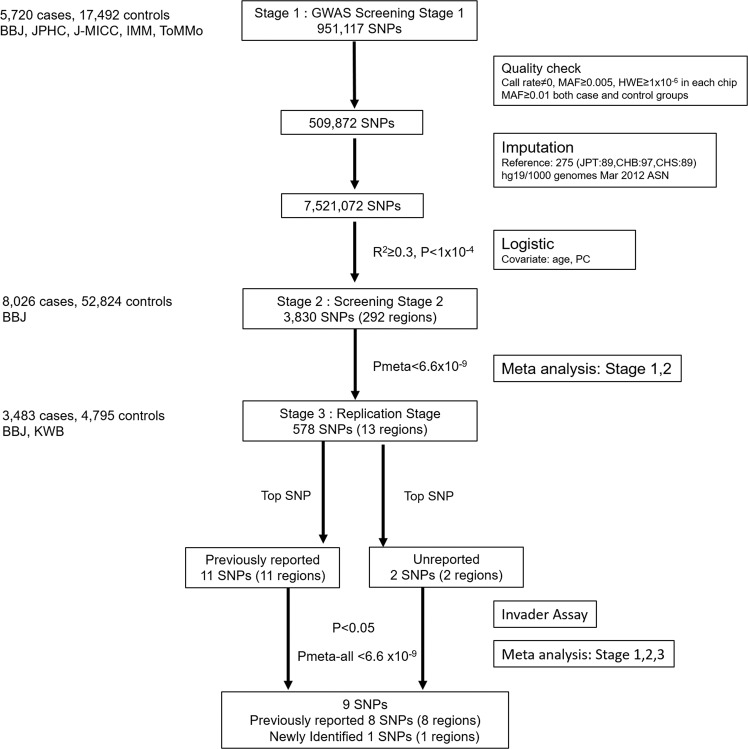
Study design of a GWAS of uterine leiomyoma. The meta-analysis comprised three stages.

**Figure 2:**
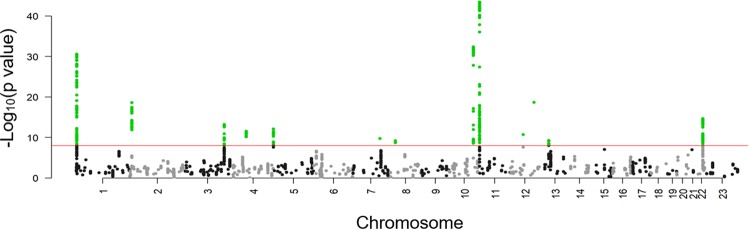
Manhattan plot of the meta-analysis of GWAS screening stage 1 and screening stage 2. The red horizontal line represents the genome-wide significance threshold of P = 6.6 × 10^−9^.

We selected 3,830 SNPs in 292 genomic regions with a suggestive association (P < 1.0 × 10^−4^) for further analysis in screening stage 2 (Fig. 1). DNA samples from 8,026 uterine leiomyoma patients and 52,824 controls that were genotyped in a previous analysis^25^ were used in stage 2. A Manhattan plot of the meta-analysis (Stage1 and 2) is shown in Fig. 2. As a result, 578 SNPs in 13 regions showed a significant association after Bonferroni correction (P < 0.05/7,521,072 = 6.6 × 10^−9^) (Supplemental Table S1).

We performed further replication analysis of SNPs in 13 regions. We selected 13 SNPs from 578 SNPs in the replication analysis using the top SNPs in each region (Fig. 1). Thirteen SNPs were successfully genotyped by the invader assay, using 3,483 cases and 4,795 controls (Supplemental Table S2). We validated the association of nine SNPs in nine genomic regions (P < 0.05) with the same risk allele as those in the GWAS (Supplemental Fig. S3).

Meta-analysis of all three stages revealed nine SNPs associated with leiomyoma risk at the genome-wide significance level (P < 6.6 × 10^−9^) (Table 1 and Supplemental Table S3). These SNPs included rs3820282 (1p36.12), rs124793436 (2p25.1), rs2251795 (3q26.2), rs2242652 (5p15.33), rs75228775 (10q24.33), rs2280543 (11p15.5), rs17033114 (12q23.2), rs7989971 (13q14.11) and rs12484776 (22q13.1). A locus on 12q23.2 (rs17033114, P = 6.12 × 10^−25^ with an OR of 1.177 (1.141-1.213)) was newly identified. Except for rs12484776 at 22q13.1, no apparent heterogeneity among the three cohorts was found (Table 1 and Supplemental Table S3). Regional plots of the nine loci are shown in Supplemental Fig. S4.

**Table 1:** Results for newly identified and previously reported risk variants associated with uterine leiomyoma at P < 6.6 × 10^−9^ in Japanese populations.

SNP	Locus	Nearest Gene	Non Reference Allele	Reference Allele	GWAS Screening Stage 1			Screening Stage 2			Replication stage			Combined				
					OR	95% CI	P	OR	95% CI	P	OR	95% CI	P	OR	95% CI	P	Heterogeneity	
																	Q^a^	I^2 b^
**Newly Identified Locus**																		
rs17033114	12q23.2	LINC00485	T	C	1.147	1.084-1.214	2.62E-06	1.185	1.135-1.237	2.25E-14	1.200	1.118-1.288	4.52E-07	1.177	1.141-1.213	6.12E-25	0.556	0.000
**Reported Loci**																		
rs3820282	1p36.12	WNT4	C	T	0.854	0.816-0.893	3.19E-12	0.853	0.825-0.882	5.42E-20	0.870	0.817-0.927	1.58E-05	0.856	0.835-0.877	3.49E-35	0.856	0.000
rs12479436	2p25.1	GREB1	G	T	1.094	1.046-1.144	6.45E-05	1.144	1.107-1.183	5.06E-15	1.072	1.006-1.141	3.09E-02	1.118	1.090-1.145	9.50E-19	0.109	54.970
rs2251795	3q26.2	MYNN	A	T	0.882	0.843-0.923	5.89E-08	0.910	0.878-0.943	9.08E-08	0.933	0.874-0.996	3.83E-02	0.904	0.882-0.928	1.24E-14	0.333	8.950
rs2242652	5p15.33	TERT	G	A	0.891	0.847-0.938	1.03E-05	0.900	0.865-0.936	1.70E-07	0.885	0.823-0.952	9.64E-04	0.895	0.870-0.921	2.42E-14	0.905	0.000
rs75228775	10q24.33	SLK	C	T	0.755	0.698-0.817	1.28E-12	0.751	0.708-0.796	2.98E-21	0.813	0.726-0.910	3.22E-04	0.761	0.729-0.795	7.45E-35	0.465	0.000
rs2280543	11p15.5	SIRT3	C	T	1.393	1.296-1.498	3.99E-19	1.337	1.266-1.412	2.15E-25	1.279	1.157-1.414	1.54E-06	1.344	1.296-1.394	2.05E-47	0.384	0.000
rs7989971	13q14.11	FLJ42392	C	G	1.122	1.073-1.174	5.14E-07	1.067	1.030-1.105	2.23E-04	1.071	1.005-1.140	3.39E-02	1.085	1.058-1.113	3.33E-10	0.207	36.590
rs12484776	22q13.1	TNRC6B	A	G	0.837	0.800-0.876	4.16E-15	0.937	0.905-0.971	2.11E-04	0.870	0.816-0.927	2.03E-05	0.893	0.871-0.916	4.63E-18	0.000	87.260

^a^Q for heterogeneity across all studies was calculated using Cochran’s Q test.

^b^I^2^, heterogeneity index.

### Association of previously reported uterine leiomyoma loci in the Japanese population

We also analysed previously reported uterine leiomyoma loci in our screening dataset. We obtained the results of 45 SNPs among 70 SNPs in 33 genomic regions (Supplemental Table S4). We found that 10 SNPs in four regions showed genome-wide significance (P < 5.0 × 10^−8^) with the same risk allele as in previous studies. In addition, 34 SNPs showed a suggestive association (P < 0.05). 35 SNPs showed same risk allele with the previous study. These results indicate that multiple variations are associated with uterine leiomyoma risk irrespective of the ethnic background.

### Association of previously reported somatic mutations in uterine leiomyoma

We also analysed SNPs on driver genes such as *MED12*, *FH*, and *HMGA2* which harbor somatic mutations in uterine leiomyoma. We obtained the results of 270 SNPs in 3 genomic regions (Supplemental Table S5). Rs4360450 nearby *MED12* at Xq13.1 was reported to be a novel candidate SNP, but this SNP is not polymorphic in the Japanese population^17^. Moreover, rs5937008 at Xq13.1 was not associated with any disease risks in our samples (P = 1.2 × 10^−1^)^15^. In addition, 19 SNPs on *MED12*, 36 SNPs on *FH*, and 215 SNPs on *HMGA2* (lowest p-value of 0.236, 0.005013, and 0.001735, respectively) did not show significant association with leiomyoma. Thus, neither of these loci were associated with uterine leiomyoma in Japanese population.

### Subgroup analysis of 9 identified loci

We conducted subgroup analysis using samples from KWB on these nine SNPs (Supplemental Fig. S5). Rs2251795, rs2242652, rs75228775, rs2280543, and rs7989971 exhibited statistically significant effects among multiple leiomyomas patients compared with single leiomyoma patients. Submucous leiomyoma causes serious symptoms such as infertility, abnormal genital bleeding, or hypermenorrhea^26^, and rs2251795 and rs75228775 were also associated with submucous leiomyoma compared with other two subtypes. Rs2280543 on 11p15.5 was associated with intramural leiomyoma^27^, but that trend was not validated in this study.

### Pleiotropic association of 9 identified loci

To further investigate the role of these loci in another hormone-related uterine hyperplastic disease (endometriosis) and other malignant tumours (endometrial cancer, ovarian cancer, breast cancer, oesophageal cancer, gastric cancer, colorectal cancer, lung cancer and prostate cancer), we analysed these SNPs in other diseases (Supplemental Fig. S6). Rs3820282, rs12479436, rs75228775, rs2280543, and rs17033114 were associated with endometriosis (P < 0.05). In addition, rs2251795 at 3q26.2 (breast, oesophageal, colorectal, lung and prostate cancer), rs2242652 at 5p15.33 (breast, gastric, lung and prostate cancer), rs75228775 at 10q24.33 (lung and prostate cancer), and rs7989971 at 13q14.11 (oesophageal and colorectal cancer) were associated with multiple cancers. Thus, six among nine loci were associated with at least one malignant disease, suggesting the role of these loci in the common neoplastic pathway.

### Association of identified loci with telomere length

The overall telomere length was significantly shorter in tumours than in adjacent matched myometrium (P = 2.35 × 10^−5^, Supplementary Fig. S7(a)), as previously reported^15,28,29^. Three regions among nine (3q26.2, 5p15.33, 10q24.33) were shown to be related to telomere length^30,31^. To evaluate the role of these genetic variations on telomere maintenance in uterine tissues, we analysed the association between variations in 3q26.2, 5p15.33, 10q24.33 and telomere length (Supplemental Table S6 and Supplemental Fig. S7(b,c)). As a result, the risk T allele of rs2251795 was significantly associated with longer relative telomere length in both normal and tumour tissues.

## Discussion

Here, we reported 9 regions that were significantly associated with uterine leiomyoma in the Japanese population. In this study, we identified 12q23.2 as a novel locus.

Although six uterine leiomyoma GWAS have been reported^14–19^, recent studies were conducted in European or African American populations. Our group previously reported a GWAS of uterine leiomyoma^18^, but more than 17,000 cases and 75,000 controls were analysed in this study. This is one of the most comprehensive uterine leiomyoma studies performed in an Asian population.

MAF of rs17033114 is about 5% and 23% in European and Japanese population. In addition, our previous study of Japanese samples only used a small sample size of 1,607 at the screening stage^18^. These differences may lead to negative association of 12q23.2 in the previous studies. rs17033114 have been reported to be related with fetal birth weight^32^, suggesting the role of this SNP in cell growth or hormonal environment in uterine. The further study is needed to clarify the molecular mechanisms how this SNP regulates fetal birth weight and uterine leiomyoma risk.

Uterine leiomyoma is known to be an oestrogen- or progesterone-dependent tumour^6,7^. Furthermore, the WNT/β-catenin signalling pathway is considered to play an important role in tumorigenesis^33–35^. On 1p36.12, rs3820282 lies in the first intron of the *WNT4*. The risk T allele of rs3820282 was shown to strengthen the binding of ERα^36^. This possible ERα/WNT4 signalling is supported by the association of the same region with endometriosis, which is an oestrogen-responsive disease. Additionally, some intronic SNPs may change the secondary structure of RNAs and DNAs, and these changes may influence gene expression and protein binding, and leads to tumorigenesis^37,38^.

Both TERT and TERC play important roles in tumorigenesis and could be involved in various malignant tumours through telomere maintainance^14^. Rs2242652 at 5p15.33 is located on *TERT*, and rs2251795 at 3q26.2 is adjacent to *TERC* (telomerase RNA component). Our findings reveal that these two SNPs were associated with some malignancies, such as breast cancer. In addition, the T allele of rs2251795 is associated with longer relative telomere length in both tumour and normal tissues (P = 0.0225 and 0.0359). Although rs2251795 has not been reported in a GWAS study, rs12638862, rs12696304, rs2293607, rs10936599, rs1317082, and rs10936601 on 3p26.2 were reported to be correlated with telomere length^39–42^. These six SNPs exhibit strong linkage disequilibrium (LD) (R^2^ > 0.71) with rs2251795 in JPT. This result is concordant with the previous studies that indicated the association of rs2293607 (T allele) and rs10936599 (C allele) (absolute linkage equilibrium with T allele of rs2251795 in JPT) with longer leukocyte telomere length^30,43^. On the other hand, the previous study reported that the risk allele of rs2736100 on TERT (5p15.33) was associated with shorter telomere length, while TERC loci (rs10936600 on 3q26.2) was not associated with telomere length in leiomyoma tissues^15^. Although the effect of uterine leiomyoma risk allele on telomere length is inverted between TERT and TERC loci, the relationship between telomere length and neoplastic disease risk is contradictory^44,45^. In addition, different SNPs on TERC and TERT loci were analysed in our and previous studies by using relatively small (less than 50) samples^15^. These results indicated the important roles of telomere pathway in the pathogenesis of uterine leiomyoma. However, further analysis is necessary to fully elucidate the functional roles of these variations on uterine leiomyoma risk.

We also observed associations of rs2251795, rs2242652, rs75228775, rs2280543 and rs7989971 with multiple leiomyomas. In addition, rs2251795 and rs75228775 were significantly related to submucous leiomyoma. Although uterine leiomyoma is benign and has many symptoms, submucous leiomyoma especially causes infertility, abnormal genital bleeding, or hypermenorrhea^26^. Collecting the information of these variants in advance may enable clinical application and personalized medicine.

There is a limitation to this study. Patients in stage 2 and stage 3 from BBJ and controls in all stages were based on medical history obtained by questionnaire or medical records. Therefore, control samples could potentially have undiagnosed leiomyoma. However, four previous GWAS studies of uterine leiomyoma also used similar criteria for control samples^14,17,18^. This possible under-diagnosis of the controls may weaken the power of the GWAS, but this should not lead to false-positive results. Because many of previously reported loci were validated in our study, we considered that our case-control samples and statistical methods/results are reliable and valid.

In conclusion, we identified nine loci, including a novel locus in the Japanese population. Our study provides evidence for the possible role of telomeres in the aetiology of leiomyoma. Our findings contribute to the elucidation of the molecular pathogenesis of uterine leiomyoma.

## Materials and Methods

### Study participants

The characteristics of each cohort are shown in Table 2. Stage 1 included 5720 leiomyoma patients from the BioBank Japan (BBJ) cohort^13,46^. Stage 2 included 8,026 patients with a history of leiomyoma from BBJ, and stage 3 included 2,582 unrelated patients with a history of leiomyoma from BBJ and 901 leiomyoma patients from Keio Women’s Health Biobank (KWB). The diagnosis of uterine leiomyoma in stage 1 was confirmed by physicians. Patients in stage 2 and stage 3 from BBJ were based on medical history obtained by questionnaire or medical records. The diagnosis of uterine leiomyoma from KWB in stage 3 was based on ultrasound examination and/or magnetic resonance imaging (MRI). The 17,492 female controls for stage 1 were from three population-based cohorts, including the JPHC (Japan Public Health Center-based Prospective) Study^47^, the J-MICC (Japan Multi-Institutional Collaborative Cohort) Study^48^, IMM (Iwate Tohoku Medical Megabank Organization) and ToMMo (Tohoku University Tohoku Medical Megabank Organization)^49^. Controls for stage 2 and 3 were from BBJ. A total of 52,824 and 4,795 female controls without a history of leiomyoma and malignancies from BBJ were used as controls for stages 2 and 3, respectively. There were no exclusion criteria, and we used all available samples at the time of the experiment. Genomic DNA was extracted from peripheral blood leukocytes using a standard method. All participants provided informed consent, and this project was approved by the ethics committees at University of Tokyo, Keio University School of Medicine, RIKEN Center for Integrative Medical Sciences, Tohoku University, Iwate Medical University, Nagoya University Graduate School of Medicine and National Cancer Center. This study was conducted in accordance with the Declaration of Helsinki.

**Table 2:** Selected characteristics of cases and controls in this study. Sample size after quality control and genotyping platforms in each of the studies included in this study.

Stage	Sample Type	Source	Number	Age Mean ± Std	Genotyping Platform
**Stage 1: GWAS screening Stage 1**					
	case	BBJ^a^	5,720	44.8 ± 9.6	OmniExpressExome BeadChip/OmniExpress and HumanExome
	control	JPHC^b^, J-MICC^c^, IMM^d^, ToMMo^e^	17,492	55.9 ± 10.0	OmniExpressExome
**Stage 2: Screening Stage 2**					
	case	BBJ^a^	8,026	64.9 ± 10.5	OmniExpressExome BeadChip/OmniExpress and HumanExome
	control	BBJ^a^	52,824	63.6 ± 15.7	
**Stage 3: Replication Stage**					
	case	BBJ^a^, KWB^f^	3,483	63.8 ± 14.4	Invader Assay
	control	BBJ^a^	4,795	65.9 ± 14.1	

^a^BBJ; BioBank Japan, ^b^JPHC; The Japan Public Health Center-based Prospective Study, ^c^J-MICC; Japan Multi-Institutional Collaborative Cohort Study, ^d^IMM; Iwate Tohoku Medical Megabank Organization, ^e^ToMMo; Tohoku University Tohoku Medical Megabank Organization, ^f^KWB; Keio Women's Health Biobank.

### Genotyping and statistical analysis

In stage 1 and stage 2, all samples were genotyped for 951,117 SNPs with the Illumina HumanOmniExpressExome BeadChip or a combination of the Illumina HumanOmniExpress and HumanExome BeadChips (Table 2) in a previous study^23^. In stage 3, samples were genotyped by the multiplex PCR-based Invader assay (Third Wave Technologies)^50^ (Table 2). In stages 1 and 2, we excluded (i) samples with a call rate < 0.98, (ii) samples from closely related individuals identified by identity-by-descent analysis, (iii) sex-mismatched samples with a lack of information, and (iv) samples from non–East Asian outliers identified by principal component analysis of the studied samples and the three major reference populations (Africans, Europeans, and East Asians) in the International HapMap Project^51^. We then applied standard quality-control criteria for variants, excluding those with (i) SNP call rate < 0.99, (ii) minor allele frequency < 1%, and (iii) Hardy–Weinberg equilibrium P value < 1.0 × 10^−6^. We prephased the genotypes with MACH^52^ and imputed dosages with minimac and the 1000 Genomes Project Phase 1 (version 3) East Asian reference haplotypes^53^. Imputed SNPs with an imputation quality R^2^ < 0.3 were excluded from the subsequent association analysis.

In stage 1, we conducted a GWAS by a logistic regression model using PLINK and incorporating the top ten principal components as covariates. A total of 3,830 SNPs (P < 1 × 10^−4^) from stage 1 were evaluated using 60,850 samples in stage 2. In stage 2, a logistic regression model incorporating age and the top two principal components was used. In stage 3, we selected 13 SNPs within 13 regions in the following two steps (Fig. 1). First, we selected the top eleven SNPs for the previously reported eleven loci. For one SNP, it was difficult to make a probe for the Invader assay, and we selected a SNP with high LD (r^2^ > 0.8) as a substitute. Second, we selected two top SNPs within the newly identified two loci.

The meta-analysis was conducted using PLINK. Heterogeneity between studies was examined using Cochran’s Q test^54^. The threshold of heterogeneity was P < 0.05/13. We calculated the genomic inflation factor λGenomic Control (GC) in R. λGC adjusted to a sample size of 1,000 (*λ*_1,000_) was calculated using the following formula^24^, as large sample sizes cause inflated λGC values^55^: *λ*_1,000_ = 1 + (1−*λ*_obs_) × (1/*n*_cases_ + 1/*n*_controls_)/(1/1,000_cases_ + 1/1,000_controls_). A quantile-quantile plot was drawn using R. A Manhattan plot of the associations was constructed by plotting −log_10_ (P values) against chromosome position using R. We generated regional plots with LocusZoom (v. 1.3)^56^. A forest plot was drawn using R.

### Subgroup analysis

We used the subjects from KWB in stage 3 for the subgroup analysis. This study analysed the clinical factors: localization, number of tumours, and recurrence. Localization and number of uterine leiomyomas were assessed using ultrasound or MRI. Leiomyomas are classified into three subgroups based on their location within the layers of the uterus. Leiomyomas are classified as subserosal (found just below the uterine serosa), intramural (within the myometrium), or submucous (located just beneath the endometrium). The numbers of leiomyomas divided into two groups: one or more than two tumours. Each subgroup was analysed using a logistic regression model with an adjustment for age. Control samples were the same as those used in stage 3. Then, comparisons between subgroups were performed using a logistic regression model with an adjustment for age.

### Pleiotropy association analysis

The association of 9 SNPs with other diseases was evaluated in the pleiotropy analysis. GWAS results in BioBank Japan for another hormone-related uterine disease (endometriosis (n = 705)) and malignant tumours (endometrial cancer (n = 931), ovarian cancer (n = 681), breast cancer (n = 5,272), oesophageal cancer (n = 1,225), colorectal cancer (n = 6,692), gastric cancer (n = 6,171), lung cancer (n = 3,874) and prostate cancer (n = 5,088))^57–59^ were used in this study.

### Tumour specimens and nucleic acid extraction

We evaluated 16 pairs of uterine leiomyoma and corresponding normal myometrium tissue, 2 normal myometrium samples and 27 uterine leiomyoma samples. These samples were collected between 2016 and 2017 at the Keio University Hospital, Tokyo, at hysterectomy or myomectomy. All tumour tissues were examined histopathologically. Tissues were preserved in RNA later solution (QIAGEN) at 4 °C until RNA extraction. Collected tissues were homogenized in QIAzol lysis reagent (QIAGEN) using Precellys 24 (Bertin Corporation). Total RNA was extracted from the tissue samples with an AllPrep DNA/RNA/miRNA Universal Kit (QIAGEN).

### Measurement of relative telomere length by real‐time quantitative PCR (qPCR)

Relative telomere length was determined by the quantitative PCR method described by Cawthon^60^. The relative telomere length was calculated as the ratio of telomere repeats to a single-copy gene (T/S ratio). The single-copy gene refers to the 36B4 gene, which encodes the acid ribosomal phosphoprotein. For each sample, the quantity of telomere repeats and the quantity of the single-copy gene were determined by comparison to a reference. The normal sample that exhibited the highest copy numbers of telomere DNA among 18 normal samples was used as a reference in Telomere length qPCR analysis. The primers used for the telomere repeat copy number and the single-copy gene copy number amplification were as follows (written 5′ → 3′): telomere forward, CGGTTTGTTTGGGTTTGGGTTTGGGTTTGGGTTTGGGTT; telomere reverse, GGCTTGCCTTACCCTTACCCTTACCCTTACCCTTACCCT; 36B4 forward, CAGCAAGTGGGAAGGTGTAATCC; 36B4 reverse, CCCATTCTATCATCAACGGGTACAA. The relative telomere length was calculated using the comparative 2^-ΔΔCt^ method: ΔΔCt = ΔCt _sample_ - ΔCt_reference sample_ and ΔCt_sample_ = Ct_Telomere_ - Ct_36B4._ The relationship between genotype and relative telomere length was examined by a linear regression analysis.

## Supplementary information

Supporting Information.

Supporting Information.

## Data Availability

The genotyping results are available in National Bioscience Database center (JGAS00000000114 / https://humandbs.biosciencedbc.jp/). Clinical information are available from Biobank Japan (https://biobankjp.org/english/index.html) on reasonable request.
