# Supplementary material for: Identification of a novel uterine leiomyoma GWAS locus in a Japanese population
Source: Sci Rep. 2020 Jan 27;10:1197. doi: 10.1038/s41598-020-58066-8 (PMC6985131; doi:10.1038/s41598-020-58066-8)

SUPPLEMENTARY INFORMATION

Identification of a novel uterine leiomyoma GWAS locus in a Japanese population

Kensuke Sakai, Chizu Tanikawa, Akira Hirasawa, Tatsuyuki Chiyoda, Wataru Yamagami, Fumio Kataoka, Nobuyuki Susumu, Chikashi Terao, Yoichiro Kamatani, Atsushi Takahashi, Yukihide Momozawa, Makoto Hirata, Michiaki Kubo, Nobuo Fuse, Takako Takai-Igarashi, Atsushi Shimizu, Akimune Fukushima, Aya Kadota, Kokichi Arisawa, Hiroaki Ikezaki, Kenji Wakai, Taiki Yamaji, Norie Sawada, Motoki Iwasaki, Shoichiro Tsugane, Daisuke Aoki, Koichi Matsuda1

Supplementary Figures S1-S7

Supplemental Figure S1: Manhattan plot of the GWAS screening stage 1.

Association with uterine leiomyoma was assessed using logistic regression adjusted for 10 principal components. The x-axis represents chromosomal location, and the y-axis shows P values on a logarithmic scale. The red horizontal line represents the genome-wide significance threshold of $P=5.0 \times 10^{-8}$.

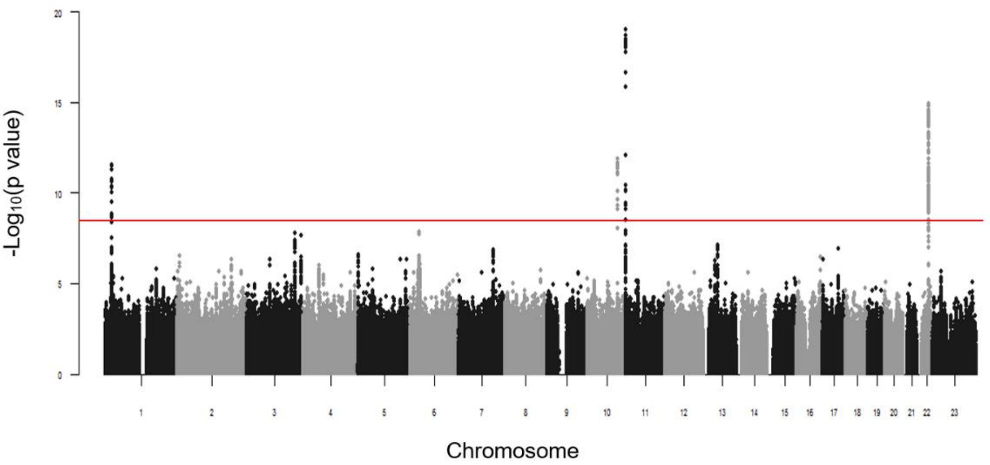

Supplemental Figure S2: Quantile-quantile (QQ) plot of the P value for GWAS screening stage 1.

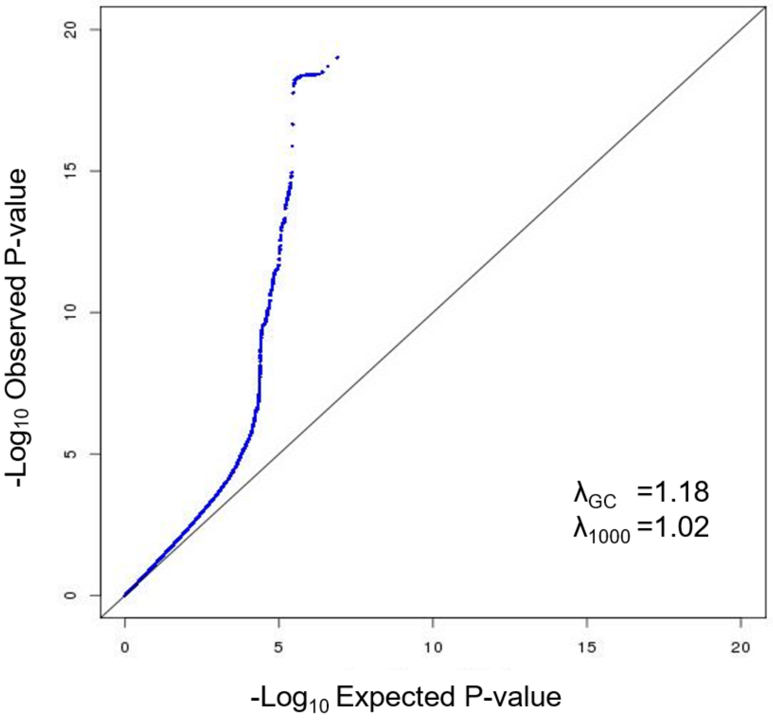

Supplemental Figure S3: Forest plots for risk variants in the 9 uterine leiomyoma risk loci. Plots show the association of odds ratios and 95% confidence intervals for GWAS screening stage 1, screening stage 2, the replication stage, and all stages combined. Squares represent the estimated per-allele odds ratio (OR) for each group. Lines indicate the 95% confidence interval.

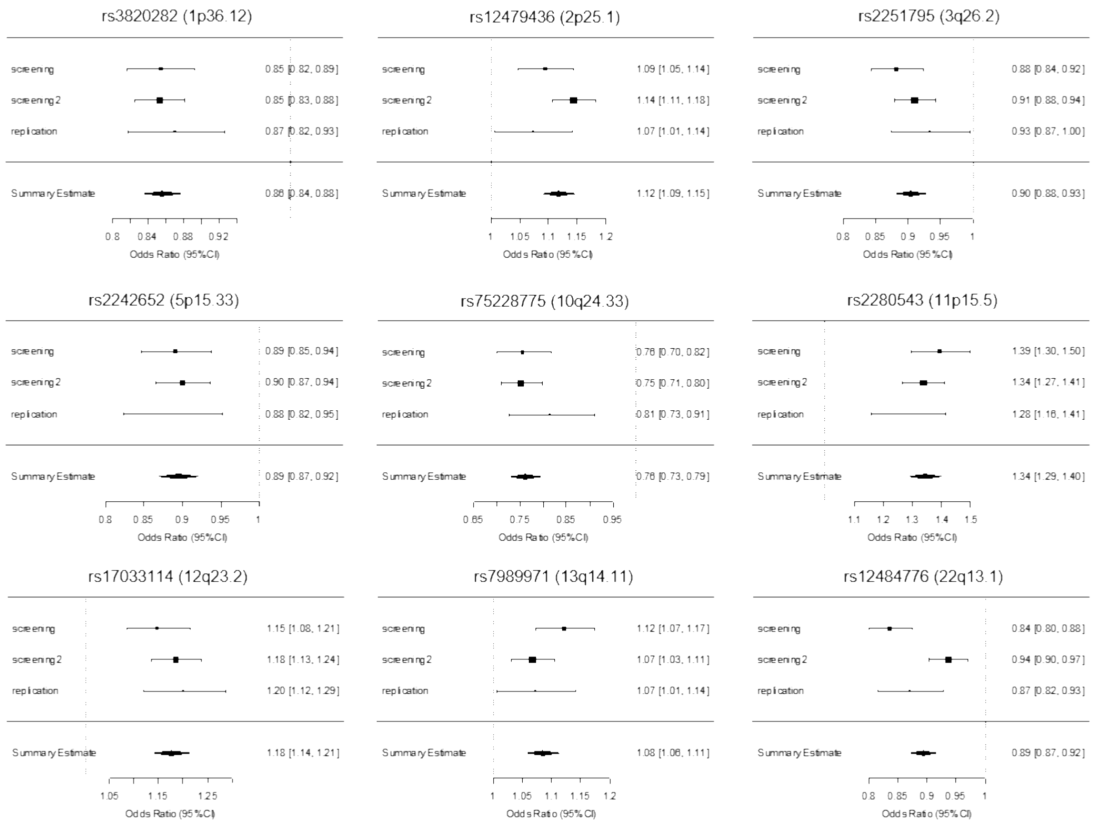

Supplemental Figure S4: Regional association plots of the risk loci.

In each plot, the log10(P values) (y-axis) for the association of SNPs with uterine leiomyoma risk are shown according to their chromosomal positions (x-axis) in NCBI Build 37. The log10(P values) from the meta-analysis of stages 1 and 2 are shown. Blue lines indicate the recombination rates from the 1000 Genomes Project (Phase 3). The linkage disequilibrium (r^2) between the marker and remaining SNPs is shown by the colour. The plots were generated using the online tool Locus Zoom[56].

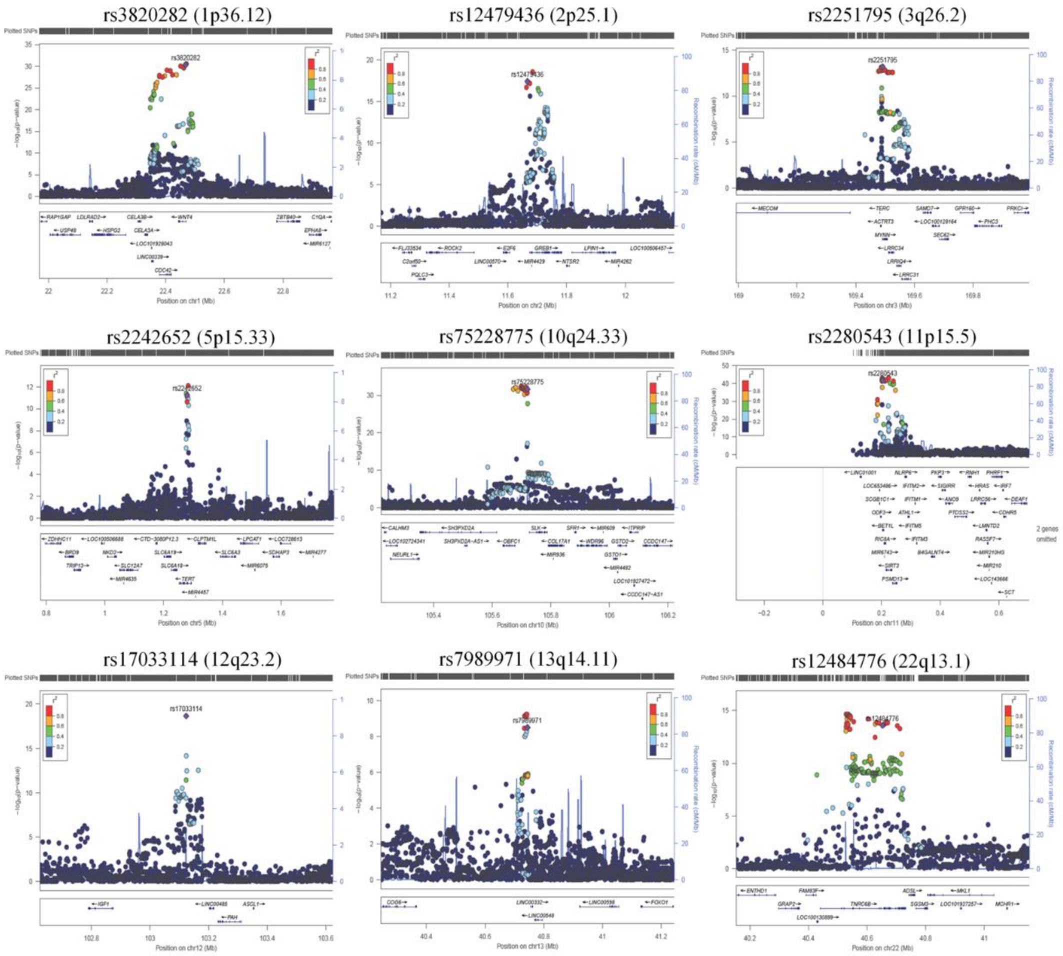

Supplemental Figure S5: Subgroup analysis of 9 uterine leiomyoma risk loci.

The association of odds ratios and 95% confidence intervals for each subgroup is shown. In this figure, all odds ratios were calculated in the replication stage. Squares represent the estimated per-allele odds ratio (OR) for each subgroup. Lines indicate the 95% confidence interval. Diamonds represent the summary OR estimates and confidence intervals for the subgroups indicated. Significant differences between two clinical subgroups are shown as asterisks (* $p < 0.05$).

KO case: KWB samples were analysed

Single: single leiomyoma cases

Multiple: multiple leiomyomas cases

Recurrent: recurrent cases

Non-recurrent: non-recurrent cases

Location_subserous: subserous leiomyoma

Location_intramural: intramural leiomyoma

Location_submucous: submucous leiomyoma

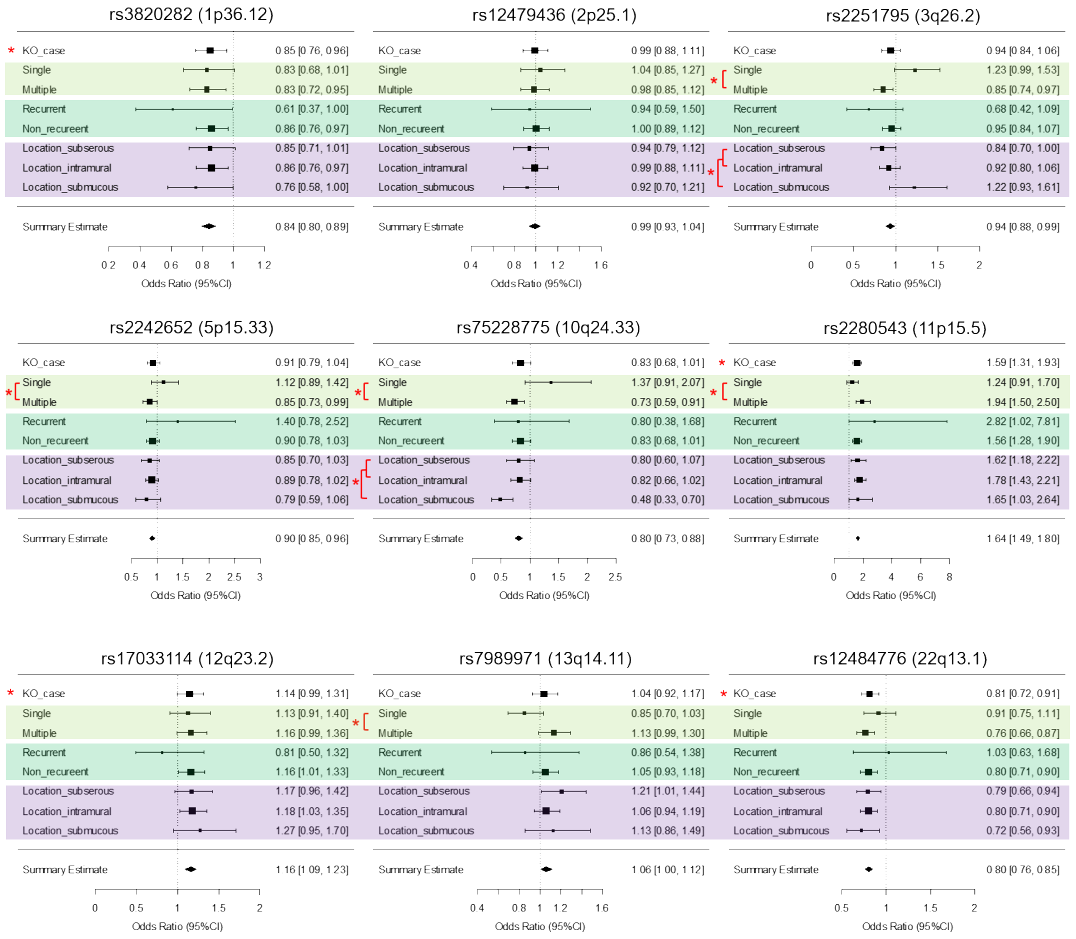

Supplemental Figure S6: Forest plots for risk variants in the 9 uterine leiomyoma loci for endometriosis or other malignant tumours.

Plots show the association of odds ratios and 95% confidence intervals for endometriosis or other malignant tumours. Squares represent the estimated per-allele odds ratio (OR) for each group. Lines indicate the 95% confidence interval. Significant differences are shown as asterisks (* p< 0.05).

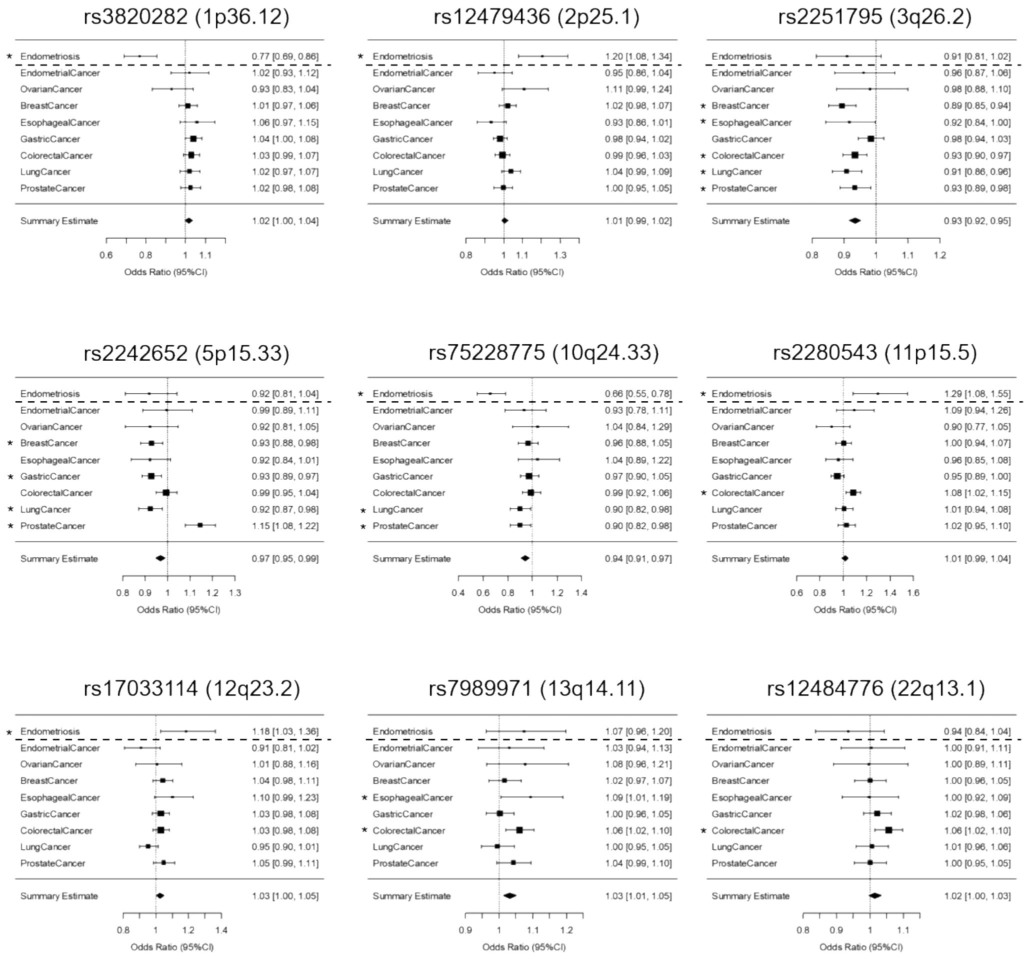

Supplemental Figure S7: Telomere length in normal myometrium tissues and tumours between genotypes of rs2251795. Note that y axis scales differ between plots.

(a) Telomere length level box plot of associations between 16 normal myometrium tissues and adjacent matched tumours.

(b) Telomere length level box plot of associations between genotypes of rs2251795 obtained from 18 normal myometrium tissues from Keio University Hospital.

(c) Telomere length level box plot of associations between genotypes of rs2251795 obtained from 43 leiomyoma tissues from Keio University Hospital.

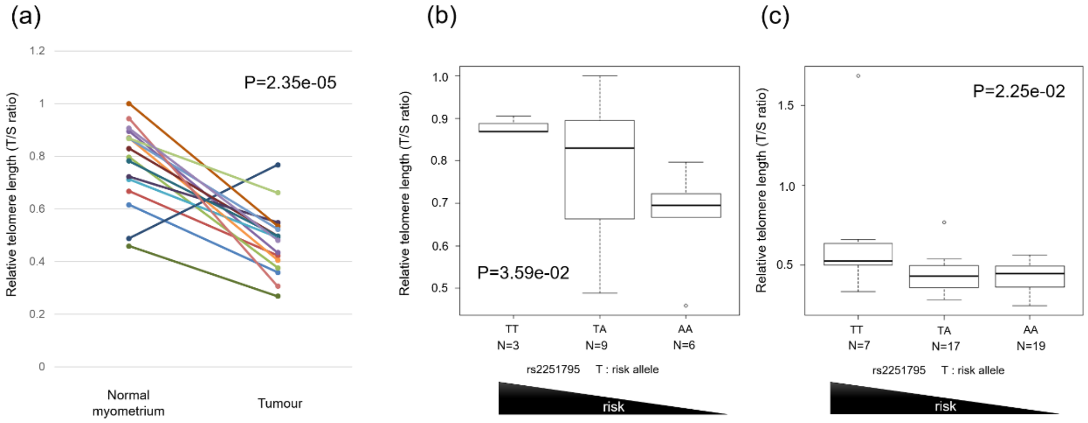

Supplement: Supplementary file 1 — Supporting Information. [file 41598_2020_58066_MOESM1_ESM.pdf]
